# Isolation and identification of alkaloids from *Macleaya microcarpa* by UHPLC–Q-TOF-MS and their cytotoxic activity in vitro, antiangiogenic activity in vivo

**DOI:** 10.1186/s13065-020-0660-1

**Published:** 2020-01-22

**Authors:** Chunmei Sai, Jian’an Wang, Binjie Li, Lin Ding, Huiyun Wang, Qibao Wang, Huiming Hua, Fangpeng Zhang, Qiang Ren

**Affiliations:** 1 College of Pharmacy, Jining Medical University, Rizhao, 276826 Shandong China; 2 Shandong Guangyu Tang Guo Yao Co., Ltd., Jining, 272071 Shandong China; 3 Key Laboratory of Structure-Based Drug Design & Discovery, Ministry of Education, Shenyang Pharmaceutical University, Shenyang, 110016 Liaoning China

**Keywords:** *Macleaya microcarpa*, Alkaloids, Isolation and identification, UHPLC–Q-TOF-MS, Biological activity

## Abstract

**Background:**

Extensive bioactivities of alkaloids from the genus *Macleaya* (*Macleaya cordata* (Willd.) R. Br. and *Macleaya microcarpa* (Maxim.) Fedde) have been widely reported, as well as more and more concerned from the scientific communities. However, systematic research on the phytochemical information of *M. microcarpa* is incomplete. The aim of this study was to rapidly and conveniently qualitative analyze alkaloids from *M. microcarpa* by ultra-performance liquid chromatography/quadrupole-time-of-fight mass spectrometry (UHPLC–Q-TOF-MS) using accurate mass weight and characteristic fragment ions, furthermore separate and identify the main alkaloids, test antitumor activity in vitro and antiangiogenic activity in vivo.

**Results:**

A total of 14 alkaloids from fruits of *M. microcarpa* were identified by UHPLC–Q-TOF-MS, including 5 protopines, 2 benzophenanthridines, 1 dimer, 1 dihydrobenzophenanthridines and 5 unknown structure compounds. Two major alkaloids were isolated by various column chromatographic methods. Their structures were determined by NMR data and related literatures. The two major alkaloids were evaluated for intro cytotoxic activities against HL-60, MCF-7, A-549, and in vivo antiangiogenic activity using transgenic zebrafish.

**Conclusions:**

Current qualitative method based on UHPLC–Q-TOF-MS technique provided a scientific basis for isolation, structural identification, and in vitro or in vivo pharmacological further study of alkaloids from *M. microcarpa* in the future.

## Introduction

The genus *Macleaya* contains two species, *Macleaya cordata* (Willd.) R. Br. and *Macleaya microcarpa* (Maxim.) Fedde, which mainly distributed in Japan, South and Northwest China, on slopes of grass or thickets at altitudes of 450–1600 m [1]. They are well-known for their very extensive application value and great exploitation foreground as well as pesticide, veterinary drug, medicinal ones in North America, Europe, China, which are directly associated with the multifarious alkaloids and their significant biological activities. A variety of alkaloids from *M. cordata*, such as sanguinarine, chelerythrine, protopine, allocryptopine, and others, exhibit anti-microbial, anti-inflammatory, insecticidal, analgesic, anticancer activity, have irreplaceable pharmacological effects [2–4]. However, systematic researches on the phytochemical composition of *M. microcarpa* are few.

UHPLC–Q-TOF-MS is widely used in qualitative compositions. The method can provide high resolution and accuracy data, as well as abundant structural information such as high-resolution second-stage mass fragment ions [5, 6]. This study aimed to rapidly and conveniently characterize alkaloids from *M. microcarpa* by UHPLC–Q-TOF-MS using accurate mass weight and characteristic fragment ions, furthermore separate and identify the main alkaloids by chromatographic and spectral techniques, test antitumor activity in vitro using the trypan blue method and MTT method reported previously [7], and antiangiogenic activity in vivo using transgenic zebrafish [8].

## Materials and methods

### Plant materials

The fruits of *Macleaya microcarpa* (Maxim.) Fedde were collected from Xiaguan Town, Neixiang County, Nanyang, Henan Province, China, in September 2017. (Notes: It was not a protective plant and was allowed to be collected). It was identified by Prof. Jian’an Wang (College of Pharmacy, Jining Medical University, Shandong, China). The voucher sample (XGBLH-20170918) was deposited in pharmaceutical experimental center, College of Pharmacy, Jining Medical University, Rizhao, China.

### Chemicals and reagents

Chromatographic grade acetonitrile was purchased from Honeywell, Burdick & Jackson. LC/MS-grade acetonitrile was purchased from Mallinckrodt Baker, Inc. (Phillipsburg, NJ, USA). Chromatographic grade formic acid was purchased from TEDIA, Inc (Fairfield, USA). Deionized water was purified by Millipore purification system (Millipore, MA, USA). Column chromatography (CC) was performed with silica gel (100–200 and 200–300 mesh, Shanghai Taitan Chemical Co. Ltd., Shanghai, China) and mci gel CHP20/P120 (Mitsubishi chemical corporation, Japan) and Sephadex LH-20 (GE Healthcare, Sweden). TLC analysis was carried out with glass plate precoated silica gel (HSGF_254_, Yantai Jiangyou Silicone Development Co. Ltd., Qingdao, China).

### Sample preparation

The air-dried and crushed fruits of *M. microcarpa* (15.0 kg) were extracted with 95% EtOH (18 L) under cold maceration 3 times, each time for 7 days, respectively. The combined extracts were concentrated in vacuo to yield crude ethanol extracts, which was suspended in H_2_O, successively partitioned with Petroleum ether (PE), Methylene chloride (CH_2_Cl_2_) and *N*-butyl alcohol (*n*-BuOH), to afford PE, CH_2_Cl_2_, *n*-BuOH and aqueous extracts (For further separation and purification).

The crude ethanol extracts (200 mg) was dissolved with 5 mL methanol by sonication at 200 W for 15 min. The solution was filtered with a 0.22 µm and then analyzed by UHPLC–Q-TOF-MS.

### UPLC–Q-TOF-MS analysis

Agilent 1290 series Rapid Resolution LC system was coupled with Agilent 6530 Accurate-Mass quadrupole time of flight (Q-TOF) mass spectrometer (Agilent Technologies, CA, USA) equipped with an electrospray ionization (ESI) interface. The chromatographic separation of analyzed crude ethanol extracts was performed on TOSOH TSK gel ODS-100V (4.6 × 150 mm, 3.0 µm) column (Tosoh Bioscience, Japan). The column temperature was maintained at 35 °C. The injection volume was 5 μL. The mobile phase consisted of 0.05% formic acid (*v/v*) (A) and acetonitrile (ACN) (B). The gradient program was applied as follows: 0–5 min at 30% B; 5–20 min at 30–54% B; 20–23 min at 54–70% B; 23–40 min at 70–80% B; 40–50 min at 80–90% B; 50–52 min at 90–30% B; 52–60 min at 30% B. The flow rate was adjusted to 1.0 mL/min. The outlet of UHPLC was split (1:4) and introduced into the ESI source.

The MS conditions were set as follows: drying gas at a flow rate of 10 L/min; drying gas temperature, 350 °C; pressure of nebulizer gas pressure, 45 psig; capillary voltage (±) 3000 V positive and negative ion modes and the mass range from *m/z* 100 to 1200 Da. The MS/MS spectra were acquired with auto MS/MS mode at the acquisition rate of 2 spectra/s.

The calculation of the elemental composition was acquired with Mass Hunter Workstation Software (Qualitative Analysis Version B.06.00) (Agilent Technologies, CA, USA).

### Isolation of two major alkaloids

The dichloromethane extract (365 g) was fractioned using silica gel column chromatography (CC) and eluted with petroleum ether (60–90 °C)–ethyl acetate (100:5, 100:10, 100:20, 100:50, 1:1 and 0:100, v/v) to yield six fractions (Fr.A–Fr.F). Fr E was subjected to Sephadex LH-20 eluting with CH_2_Cl_2_–MeOH (1:1), and further recrystallization to yield compounds **2** (prtopine, 40 mg). The precipitate during extraction of dichloromethane was separated by mci gel CHP20P CC eluting with MeOH–H_2_O (85:15) to afford compound **7** (chelerythrine, 30 mg).

### NMR spectral analysis

^1^H and ^13^C NMR spectra were acquired with Bruker AV-600 NMR spectrometer (Billerica, MA, USA) using solvent signals (CDCl_3_: *δ*_H_ 7.26/*δ*_C_ 77.16, CD_3_OD: *δ*_H_ 3.31/*δ*_C_ 49.00), with tetramethylsilane (TMS) as an internal standard.

### Cytotoxic activity test in vitro

The method of cytotoxic activity test in vitro had based on our previously published work [9]. HL-60 (human leukaemia cell lines), MCF-7 (human breast cancer cell lines), A-549 (human lung adenocarcinoma cell lines), which were purchased from America Type Culture Collection, ATCC (Rockville, MD, USA) and cultured in RPMI-1640 medium (Gibco, New York, NY, USA) supplemented with 100 U/mL penicillin, 100 mg/mL streptomycin, 1 mM glutamine and 10% heat-inactivated fetal bovine serum (Gibco) at 37 °C in humidified atmosphere with 5% CO_2_. Compounds **2** and **7** were evaluated for cytotoxic activities by the trypan blue method against HL-60, and MTT assay against MCF-7 and A-549 [10, 11].

In the trypan blue method, cells in logarithmic growth were seeded at 5 × 10^4^ cells/mL in 24-well microplates, 2 mL/well, and incubated with various concentrations of the compounds at 37 °C for 72 h. 50 μL suspension was taken from each well, and 50 μL 0.4% trypan blue was added to mix well, and observed under an optical microscope within 3 min. Trypan blue-stained (nonviable) cells and the total cell number were determined with a hematocytometer. The growth inhibition in cells after treatment with different concentrations was calculated comparing with control cells (5-Fluorouracil was used as a positive control), and a half growth inhibitory concentration (IC_50_) was obtained by regression analysis of the concentration response data.

In the MTT assay, briefly, cells suspensions, 100 μL, at a density of 2.5 × 10^4^ cells/mL, were plated in 96-well microtiter plates and incubated for 24 h at 37 °C. Then the test compounds with different concentrations in DMSO, 100 μL, were placed into each microtiter plates and further incubated for 72 h. Finally, 50 μL of a 0.4% MTT solution was added to each well and incubated for 4 h. Then, the MTT was removed from the wells and the formazan crystals were dissolved in DMSO (200 μL) for 10 min with shaking. Then the plate was read immediately on a microtiter plate reader (Bio-RAD) at a wavelength of 570 nm to record the optical density (OD). The IC_50_ value was defined as the concentration of the control in the MTT assay. 5-Fluorouracil (5-Fu) was used as a positive control. All the IC_50_ results were expressed as average of three independent experiments.

### Antiangiogenic activity test in vivo

Transgenic Tg (flk: EGFP) zebrafish was provided by drug screening laboratory, Biology Institute of Shangdong Academy of Sciences. PTK787 was provided by biochemistry laboratory, Biology Institute of Shangdong Academy of Sciences (20110902).

Angiogenesis plays a critical role in cancer growth and metastasis. Antiangiogenesis is an excellent target in cancer treatment. In recent assays, the zebrafish model is practical and efficient in vivo model in screening natural product and drug for anti-angiogenesis. [12, 13].

### Zebrafish embryo collection

Male and female transgenic Tg (flk: EGFP) zebrafish were fed separately, and regularly fed with artificial pellet bait and newly hatched *Artemia nauplii*, stocks were maintained in a controlled environment at 28.5 °C on a 14 h: 10 h light/dark cycle. Healthy sexual mature zebrafish were put into the same mating cylinder in 1:1 ratio of male and female. Fertilized eggs were obtained at am 9–10 the following day. After disinfection and washing, the fertilized eggs were transferred to embryo water (containing 5.0 mM NaCl, 0.17 mM KCl, 0.4 mM CaCl_2_, 0.16 mM MgSO_4_), and cultured at 28 °C.

### Zebrafish embryo antiangiogenesis assay

Healthy and limpid embryos were picked out at 24 h post-fertilization (hpf) and distributed into a 24-well microplate (6–8 embryos/well) in 1 mL Holtfreter’s solution and maintained at 28 °C. The sample solution was diluted with embryo water to different concentrations of 0.5, 1, 10, 100 μg/mL, and added into the well. The final volume of each well was 2.0 mL, and the content of DMSO in each well was adjusted to be consistent. 2.0 mL 0.1 μg/mL PTK787 solution served as positive controls. The embryo water or DMSO (0.5%, V/V) served as blank controls. Embryos were maintained in incubator at 28 °C for additional 48 h, placed onto a glass slide, photographed using SZX16 fluorescence stereomicroscope and DP2-BSW image acquisition system (Olympus, Japan) after anesthesia. Zebrafish somite intersegmental vessels (ISVs) were quantified using Image Pro Plus software. Anti-angiogenic effects were defined as decrease of SIVs length [14].

### Statistical analysis

SPSS 13.0 was used for statistical analysis, and independent sample t test was used to compare the differences among the groups. P < 0.05 was considered as significant difference.

The qualifications and experience of the researcher met the experimental requirements, after the review by the ethics committee of Jining Medical University, and the research design conformed to the principles of scientific science and medical ethics (Ethical code 2019-YX-256).

## Results and discussion

### Analysis of alkaloids from fruits of *M. microcarpa* by UHPLC–Q-TOF-MS

**Fig. 1:**
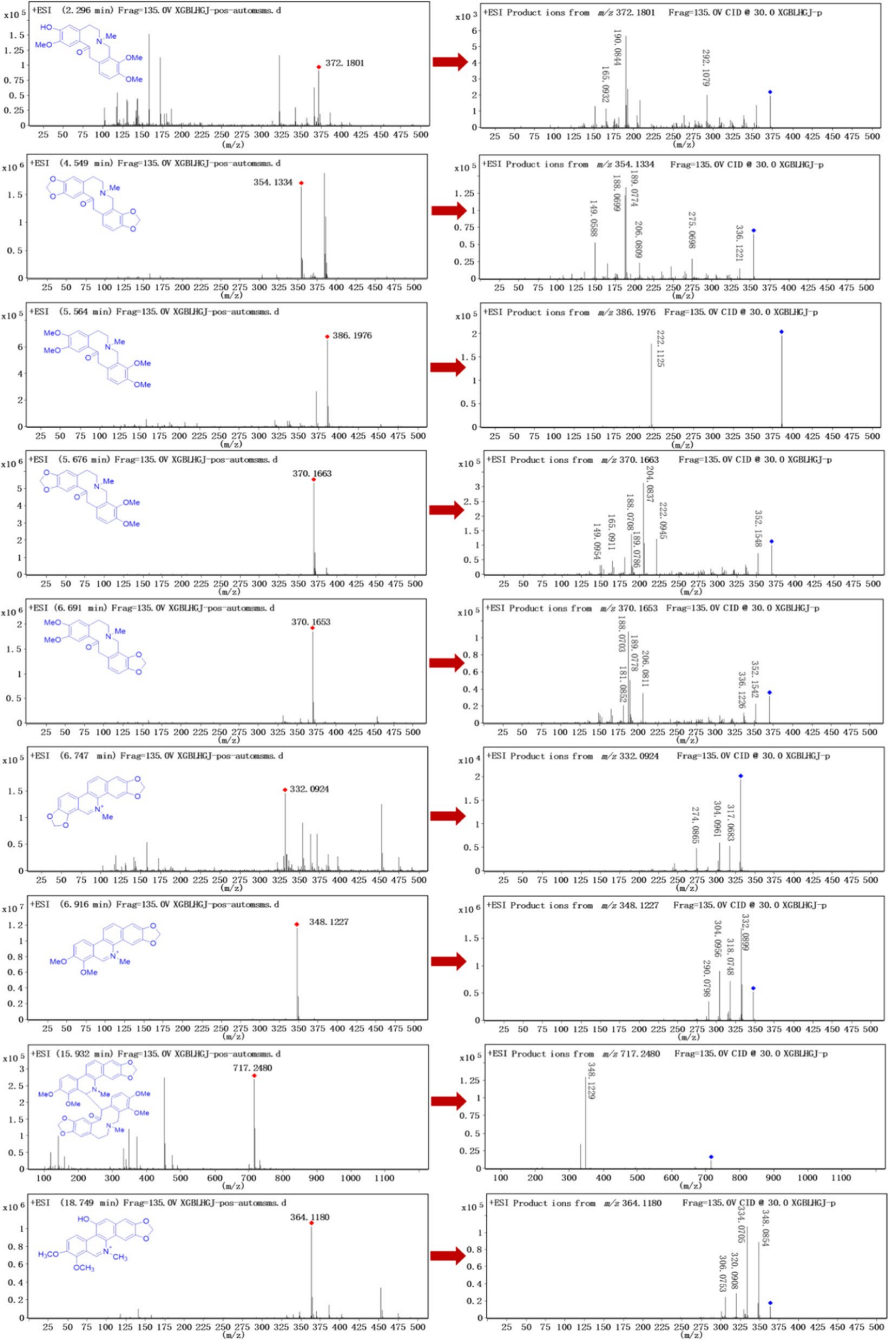
The high resolution mass spectra and secondary mass spectra for 9 known compounds

**Fig. 2:**
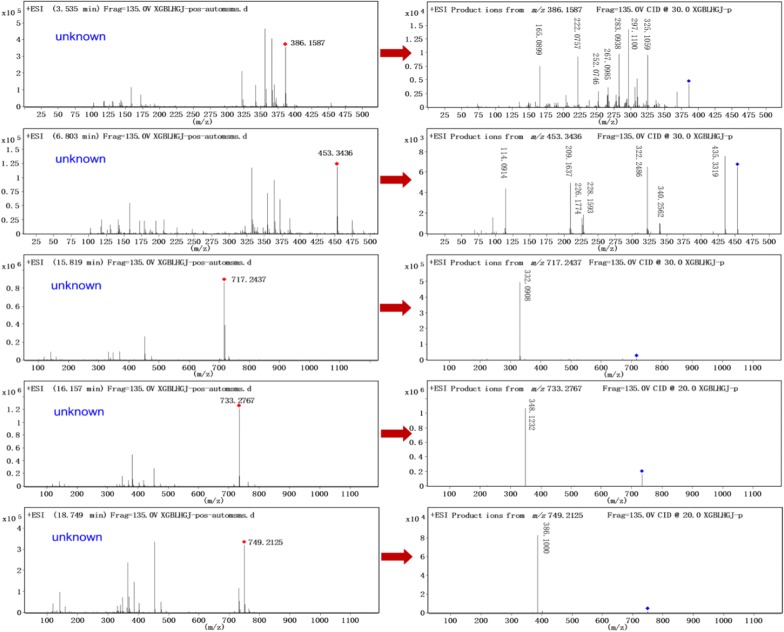
The high resolution mass spectra and secondary mass spectra for 5 unknown compounds

**Fig. 3:**
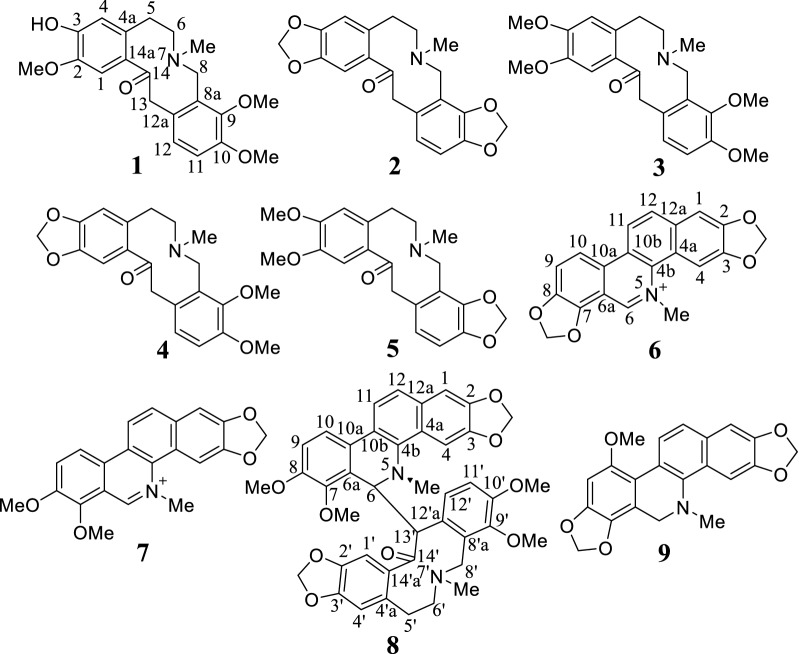
Structures of compounds **1**–**9**

**Fig. 4:**
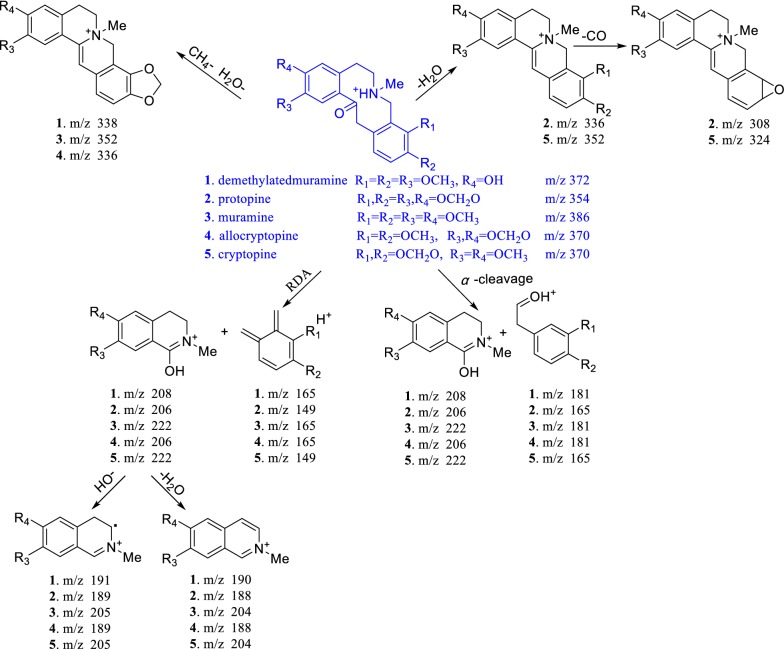
Mass spectral fragmentation behavior of protopine alkaloids

**Fig. 5:**
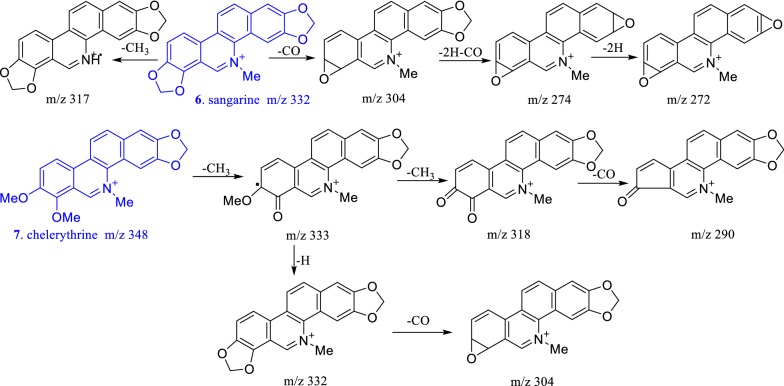
Mass spectral fragmentation behavior of benzophenanthrine alkaloids

**Fig. 6:**
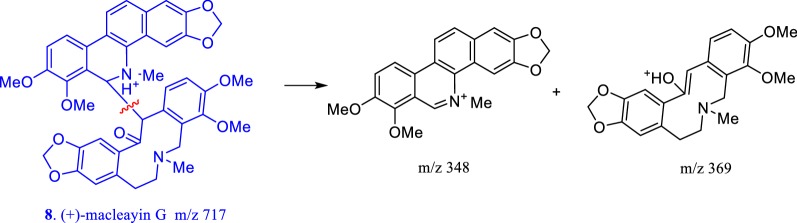
Mass spectral fragmentation behavior of dimer alkaloids

**Fig. 7:**
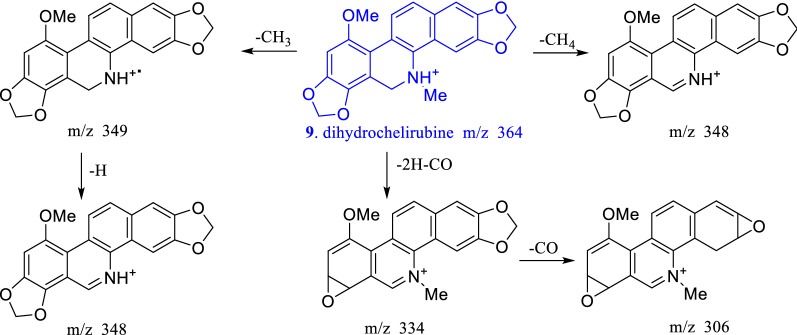
Mass spectral fragmentation behavior of dihydrobenzophenanthrine alkaloids

In this study, UHPLC–Q-TOF-MS technology was used to qualitative analyzed and identified alkaloids from fruits of *M. microcarpa*. The high resolution mass spectra and secondary mass spectra are shown in Figs. 1 and 2. These compositions are summarized along with their retention time, theoretical mass, molecular formula, observed mass, error and MS/MS fragments combined with literature reports [15, 16] and previously isolated alkaloids from *M. cordata* [7, 9, 17, 18]. A total of 14 possible alkaloids have been identified, of which 9 are known. The detailed data are listed in Table 1 and Fig. 3. The mass spectral fragmentation behaviors of 9 known and identified alkaloids are shown in Figs. 4, 5, 6 and 7.

**Table 1:** UHPLC–Q-TOF-MS data of identified alkaloids from fruits of *M. microcarpa*

Peak no.	*T*_R_ (min)	Theoretical mass (*m/z*)	Molecular formula	Error (ppm)	Observed mass (*m/z*)	Fragment ions of MS^2^ (*m/z*)	Identified compounds
1	2.296	372.1805	C_21_H_25_NO_5_	1.21	372.1801	165 (20), 181 (10), 190 (100), 191 (24), 192 (42), 208 (29), 338 (12), 354 (23), 372 (35)	Demethylatedmuramine
2	4.549	354.1336	C_20_H_19_NO_5_	0.56	354.1334	149 (39), 165 (17), 188 (90), 189 (100), 190 (7), 206 (17), 336 (11)	Protopine
3	5.564	386.1962	C_22_H_27_NO_5_	− 3.64	386.1976	222 (91), 386 (100)	Muramine
4	5.676	370.1649	C_21_H_23_NO_5_	− 3.79	370.1663	149 (10), 165 (15), 188 (34), 189 (44), 190 (9), 204 (100), 222 (37), 352 (22)	Allcryptopine
5	6.691	370.1649	C_21_H_23_NO_5_	− 1.09	370.1653	165 (15), 181 (19), 188 (100), 189 (47), 190 (9), 206 (32), 336 (11), 352 (21)	Cryptopine
6	6.747	332.0923	C_20_H_14_NO_4_	− 0.35	332.0924	274 (24), 304 (30), 317 (27), 332 (100)	Sanguinarine
7	6.916	348.1236	C_21_H_18_NO_4_	2.54	348.1227	290 (20), 304 (53), 318 (42), 332 (100), 333 (39), 348 (31)	Chelerythrine
8	15.932	717.2807	C_42_H_40_N_2_O_9_	1.34	717.2797	348 (100), 349 (5)	(±)-Macleayin G
9	18.749	364.1185	C_21_H_18_NO_5_^+^	1.37	364.1180	306 (23), 320 (26), 334 (100), 348 (16), 349 (82), 364 (12)	Dihydrochelirubine
10	3.535				386.1587	165 (52), 204 (15), 222 (65), 252 (20), 266 (16), 267(25), 283 (68), 306 (26), 309 (37), 325 (66), 386 (29)	Unknown
11	6.803				453.3436	435 (100), 114 (58), 209 (65), 226 (20), 227 (10) 228 (24), 322 (86), 340 (13), 453 (86)	Unknown
12	15.819				717.2437	332 (100), 717 (2)	Unknown
13	16.157				733.2767	348 (100), 733 (15)	Unknown
14	18.749				749.2125	386 (100), 749 (2)	Unknown

The mass spectral cleavage behaviors of protopine alkaloids had the following rules (Fig. 4): The parent nucleus lost one molecule of H_2_O to form a closed four-membered ring. Protopine alkaloids did not contain large π conjugate system, and the parent nucleus was prone to happen RDA cracking and *α* cleavage reactions, forming small fragment molecules, which would continue to lose hydroxyl or H_2_O moiety to form fragment peaks of [M_A_–17]^+^ or [M_A_–18]^+^, respectively. The mass spectral cleavage rules of benzophenanthridine alkaloids were as follows (Fig. 5): Benzophenanthridine alkaloids were large π conjugate system, and the parent nucleus was difficult to fragment. If benzophenanthridine alkaloids contain methylenedioxyl, they would lose carbon monoxide to form a stable ternary oxygen ring. If *O*-dimethoxy group was present in benzophenanthridine alkaloids, the *O*-dimethoxy group would first lose a methyl moiety and then lose hydrogen to form a methylenedioxyl, and the methylenedioxyl will continue to lose one carbon monoxide to form a stable ternary oxygen ring. If benzophenanthridine alkaloids contain 5- or 6-methoxy or methyl groups, the methoxy or methyl groups would be directly lost to form [M-31]^+^ or [M-15]^+^ mass spectral fragments. The cleavage of the dimer mainly occurs at the junction of two alkaloids, to form corresponding precursory alkaloids (Fig. 6).

According to this cleavage rule and the MS/MS fragment peaks, compound **12** might be a dimer formed by sanguinarine and muramine by the C–C single bond, compound **13** might be a dimer formed by chelerythrine and muramine by the C–C single bond, compound **14** might be a dimer formed by muramine and a new benzophenanthridine alkaloid by the C–C single bond. To search the related molecular formula from the Scifinder database, **12**–**14** would be novel compounds. Their exact structures would be determined by NMR after separation and purification. Dihydrobenzophenanthridine alkaloids first lost 6- or 5-substituents to form relatively stable benzophenanthridine alkaloids, and the following fragment rule was consistent with that of benzophenanthridine alkaloids (shown in Fig. 7). The cleavage rules were helpful to identify the characteristic alkaloids in *M. microcarpa* by LC–MS method.

### NMR data of compounds 2 and 7

Two major alkaloids, protopine and chelerythrine, were isolated and prepared from *M. microcarpa* for later activity testing.

Protopine (**2**) was isolated as colorless square crystal in CH_2_Cl_2_: MeOH (1:1). ^1^H NMR (400 MHz, CDCl_3_) *δ*: 6.90 (1H, s, H-1), 6.69 (1H, d, *J* = 7.8 Hz, H-12), 6.66 (1H, d, *J* = 7.8 Hz, H-11), 6.64 (1H, s, H-4), 5.95 (2H, s, –OCH_2_O-2,3), 5.92 (2H, s, –OCH_2_O-9,10), 3.78 (2H, br s, H-13), 3.58 (2H, br s, H-8), 2.2–3.2 (4H, br s, H-5, 6), 1.91 (3H, s, *N*-CH_3_). ^13^C NMR (100 MHz, CDCl_3_) *δ*: 108.3 (C-1), 146.5 (C-2), 148.1 (C-3), 110.6 (C-4), 132.9 (C-4a), 31.9 (C-5), 57.9 (C-6), 50.9 (C-8), 118.0 (C-8a),146.0 (C-9), 146.1 (C-10), 106.9 (C-11), 125.2 (C-12), 129.1 (C-12a), 46.6 (C-13), 195.1 (C-14), 136.3 (C-14a), 101.3 (–OCH_2_O-2,3), 101.0 (–OCH_2_O-9,10), 41.6 (*N*-CH_3_). The structure was identified by comparison of the NMR data with literature [19].

Chelerythrine (**7**) was yellow powder. ^1^H NMR (400 MHz, CD_3_OD) *δ*: 9.99 (1H, s, H-6), 8.71 (1H, d, *J *= 9.0 Hz, H-10), 8.68 (1H, d, *J* = 9.2 Hz, H-11), 8.24 (1H, d, *J* = 9.0 Hz, H-9), 8.23 (1H, d, *J* = 9.2 Hz, H-12), 8.21 (1H, s, H-4), 7.59 (1H, s, H-1), 6.28 (2H, s, –OCH_2_O-2,3), 4.30 (3H, s, 7-OCH_3_), 4.15 (3H, s, 8-OCH_3_), 5.01 (3H, s, *N*-CH_3_). ^13^C NMR (100 MHz, CD_3_OD) *δ*: 107.1 (C-1), 151.8 (C-2), 150.8 (C-3), 105.1 (C-4), 121.9 (C-4a), 132.6 (C-4b), 52.9 (*N*-CH_3_), 152.1 (C-6), 119.9 (C-6a), 147.6 (C-7), 151.8 (C-8), 127.5 (C-9), 121.0 (C-10), 130.2 (C-10a), 127.2 (C-10b), 119.5 (C-11), 132.7 (C-12), 134.4 (C-12a), 104.3 (–OCH_2_O-2,3), 62.8 (7-OCH_3_), 57.6 (8-OCH_3_). According to related literatures [20, 21] and NMR data, the compound was determined as chelerythrine.

### Cytotoxic activity in vitro

Compounds **2** and **7** exhibited potent cancer cell growth inhibitory activities against HL-60, A-549, and MCF-7 cancer cell lines. The results are summarized in Table 2.

**Table 2:** In vitro antiproliferative activities

Compounds	HL-60 IC_50_ (μm)	A-549 IC_50_ (μm)	MCF-7 IC_50_ (μm)
Protopine	6.68	20.47	22.59
Chelerythrine	6.68	5.37	5.26
5-Fu	3.10	1.80	16.68

### Antiangiogenic activity in vivo

**Fig. 8:**
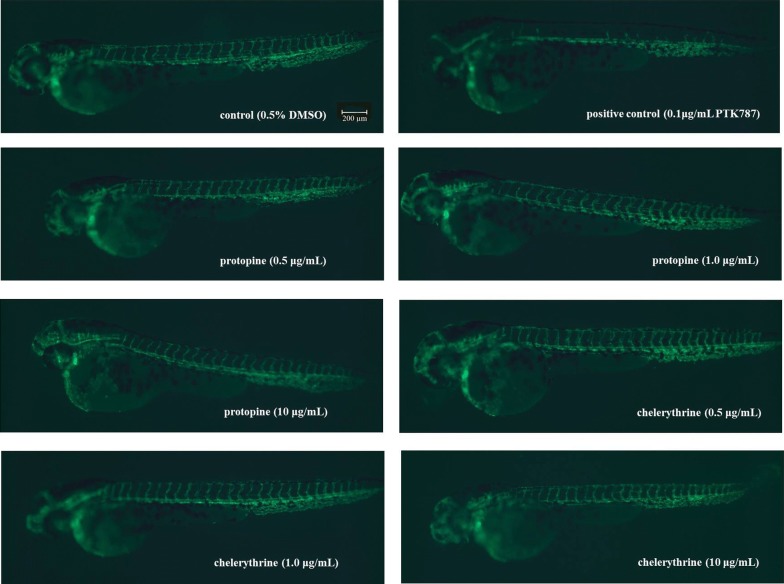
In vivo antiangiogenic effects of protopine and chelerythrine in transgenic zebrafish

In order to confirm that protopine and chelerythrine were associated with inhibition of tumor angiogenesis, we used a transgenic zebrafish model to evaluate the antiangiogenesis effects. Transgenic zebrafish embryos at 24 hpf treated with protopine and chelerythrine for 48 h showed a dose-dependent inhibition of ISV formation. The inhibitory effect of different doses of sample on intersegmental vessel (ISV) formation in zebrafish embryos were shown in Fig. 8 and Table 3. But there were no significant difference in the total length and morphology of ISVs between the treatment group and the control group. All the young fish in each 100 μg/mL sample group died. From these results, we could speculate that the antitumor effects of the samples were not achieved by inhibiting the vascular growth mechanism.

**Table 3:** Total length of ISV of zebrafish in different treatment groups

Group	Total length of ISV (μm)	*P*
Control (0.5% DMSO)	3021.07 ± 359.8	–
Positive control (0.1 μg/mL PTK787)	700.08 ± 214.2^**^	0.006
Protopine (0.5 μg/mL)	2946.44 ± 403.7	0.880
Protopine (1 μg/mL)	2950.27 ± 378.9	0.881
Protopine (10 μg/mL)	2818.69 ± 407.4	0.688
Chelerythrine (0.5 μg/mL)	2952.11 ± 489.4	0.903
Chelerythrine (1 μg/mL)	3065.54 ± 304.9	0.915
Chelerythrine (10 μg/mL)	3091.49 ± 525.6	0.906

**Comparison to control group *P *< 0.05

## Conclusions

In this paper, we rapidly and conveniently qualitative analyze alkaloids from *M. microcarpa* by UHPLC–Q-TOF-MS using accurate mass weight and characteristic fragment ions, and combining with their cleavage rules. Some unknown compounds were discovered through this simple and sensitive method. The study on the cleavage rules of these alkaloids is helpful to identify the characteristic alkaloids by LC–MS method, and identify the structural types of alkaloids in *M. microcarpa*. The two major alkaloids of *M. microcarpa*, protopine and chelerythrine, exhibited potent cancer cell growth inhibitory activities in vitro, but they showed almost no antiangiogenic activity in transgenic zebrafish vivo model. However, the structure and bioactivity screening of new natural products from *M. microcarpa* still need further study, which is an integral part of drug discovery progress.

## Data Availability

The datasets used and/or analysed during the current study are available from the corresponding author on reasonable request.

## Abbreviations

UHPLC–Q-TOF-MS: ultra high performance liquid chromatography/quadrupole-time-of-fight mass spectrometry
NMR: nuclear magnetic resonance
HL-60: the human leukaemia cell line
MCF-7: the human breast cancer cell line
A-549: the human lung adenocarcinoma cell line
MTT: 3-(4,5-dimethylthiazol)-2,5-diphenyltetrazolium bromide
LC/MS: liquid chromatograph/mass spectrometer
TLC: thin layer chromatography
LC: liquid chromatograph
UHPLC: ultra high performance liquid chromatography
ESI: electrospray ionization
MS: mass spectrometry
IC_50_: the concentration of drug required to inhibit cell growth by 50% compared with untreated control
ISVs: intersegmental vessels
*m/z*: mass-to-charge ratio
